# Severe Respiratory Diphtheria-Like Illness Caused by Toxigenic *Corynebacterium ulcerans*

**DOI:** 10.3201/eid3202.250908

**Published:** 2026-02

**Authors:** Rita Helleren, Hans Kristian Fløystad, Odd Alexander Tellefsen, Miroslav Boskovic, Terje Skraastad, Anne Torunn Mengshoel, Kristian Alfsnes, Vegard Skogen

**Affiliations:** Sørlandet Hospital, Kristiansand, Norway (R. Helleren, H.K. Fløystad, O.A. Tellefsen, M. Boskovic, T. Skraastad); Norwegian Institute of Public Health, Oslo, Norway (A.T. Mengshoel, K. Alfsnes); Faculty of Health Sciences, UiT The Arctic University of Norway, Tromsø, Norway (V. Skogen); University Hospital of North Norway, Tromsø (V. Skogen)

**Keywords:** Corynebacterium ulcerans, bacteria, bacteremia, diphtheria, zoonoses, respiratory infections, Norway

## Abstract

We report a possible zoonotic case of severe diphtheria-like respiratory illness in Norway caused by a previously unreported toxigenic *Corynebacterium ulcerans* sequence type. This case highlights *C. ulcerans* as an emerging pathogen that can cause life-threatening disease. Clinicians should be aware of *C. ulcerans* infection, even in regions where diphtheria is rare.

*Corynebacterium ulcerans* bacteria can cause respiratory and nonrespiratory infections in humans, and respiratory diphtheria-like illness caused by toxigenic *C. ulcerans* is increasing (*1*). Globally, *C. ulcerans* bacteremia is rare, and limited clinical data are available. We describe a case of severe respiratory diphtheria-like illness caused by toxigenic *C. ulcerans* bacteremia in an immunocompromised patient in Norway without travel history.

On January 11, 2024, a 74-year-old immunocompromised man was referred to the emergency department for a 2-day history of breathlessness, productive cough, malaise, and cognitive impairment. He had a sore throat and cold-like symptoms 8–9 days earlier. He lived in an urban area with his wife and dog. Medical history included coronary disease, type 2 diabetes mellitus, chronic obstructive pulmonary disease with obstructive sleep apnea, and a hemicolectomy for cancer in 2019. He had been immunocompromised since 2021 from weekly methotrexate (20 mg) and etanercept (50 mg) for psoriatic arthritis.

At admission, he was afebrile but in respiratory distress with marked hoarseness and wheezing on auscultation; throat examination was unremarkable. He was hemodynamically stable; oxygen saturation was 88% on room air. Electrocardiograph showed sinus rhythm; echocardiography revealed preserved biventricular systolic function. Chest radiograph showed a retrocardiac infiltrate; laboratory findings indicated infection (Table).

**Table:** Laboratory findings from blood collected at admission in a case of severe respiratory diphtheria-like illness caused by toxigenic *Corynebacterium ulcerans*, Norway*

Test	Value	Reference range
C-reactive protein, mg/L	329	0.00–5.0
Leukocytes, × 10^9^ cells/L	24	3.5–10.0
Neutrophils, × 10^9^ cells/L	22	1.5–7.1
Creatinine, µmol/L	183	60–105
GFR, mL/min	31	>90

*GFR, glomerular filtration rate.

He was treated with ampicillin (1 g 4×/d), prednisolone (40 mg), inhaled bronchodilators for suspected exacerbation of his pulmonary disease, and 2 L/min of oxygen via nasal cannula. Blood cultures taken at admission showed gram-positive rods after 27 hours using the BD Bactec FX system (Becton Dickinson, https://www.bd.com). MALDI BioTyper (Bruker Daltonik GmbH, https://www.bruker.com) identified *C. ulcerans*, prompting a diphtheria-like illness diagnosis. The Norwegian Institute of Public Health confirmed *C. ulcerans* using API Coryne version 4.0 system (bioMérieux, https://www.biomerieux.com) and by PCR. We also identified the *tox* gene by PCR and toxin production by a modified Elek test (*2*). We sequenced the isolate on a NextSeq platform (Illumina, https://www.illumina.com), and curators at BIGSdb-Pasteur MLST database (https://bigsdb.pasteur.fr) identified it as sequence type 1032.

Two days after admission, antibiotic therapy was changed to intravenous penicillin G ( 3 g 4×/d), and the patient received 100,000 units of diphtheria antitoxin. Respiratory distress worsened, and the patient was transferred to the intensive care unit. Four days after admission, ventricular tachycardia developed but was successfully cardioverted after intubation.

The patient required mechanical ventilation for 7 days. Bronchoscopy revealed pseudomembranes in the lower airways, which were removed (Figure). He completed 14 days of antibiotic therapy, mainly benzylpenicillin, and 7 days of cefotaxime for ventilator-associated *Staphylococcus aureus* pneumonia.

**Figure:**
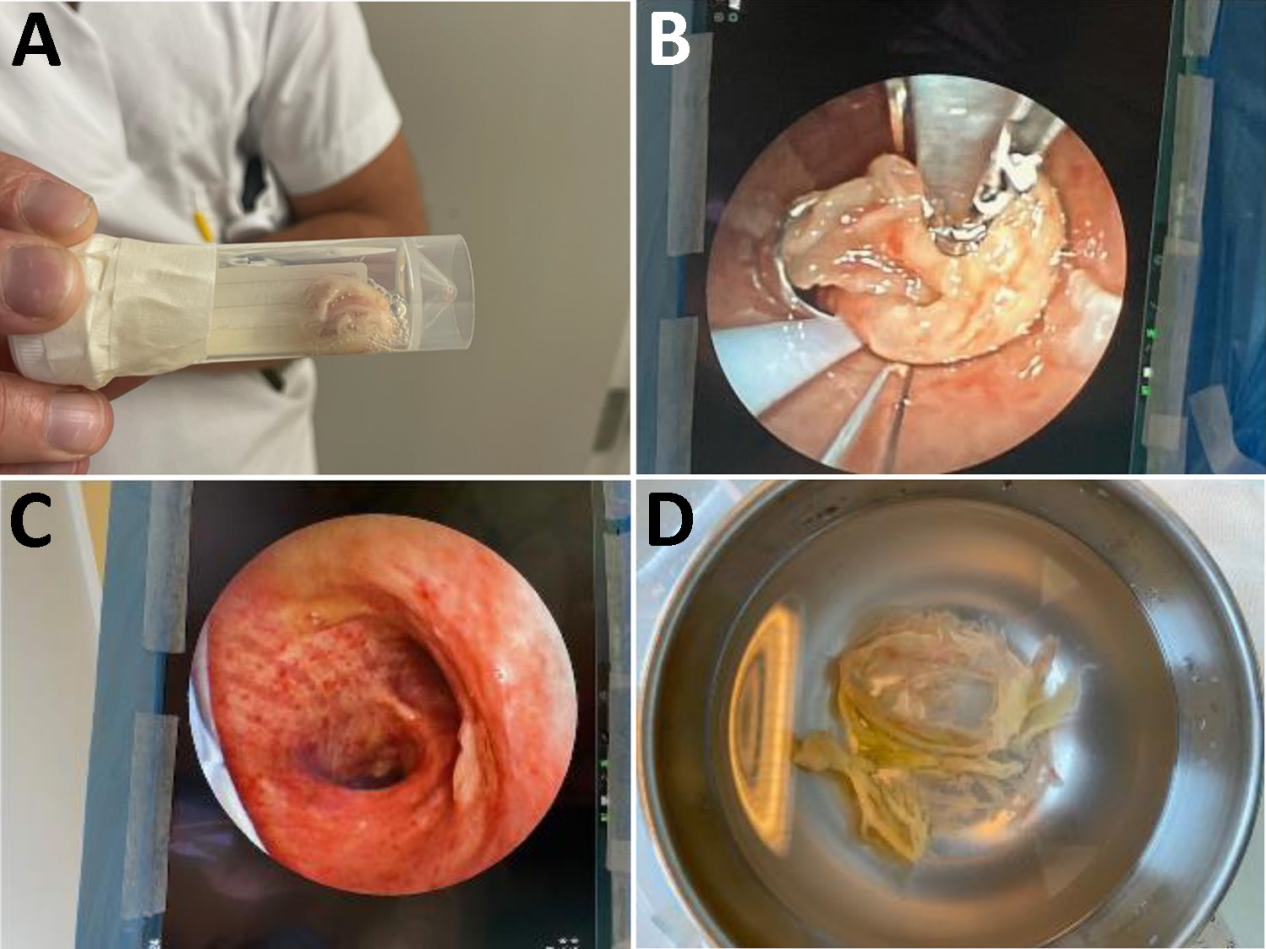
Clinical findings from a case of severe respiratory diphtheria-like illness caused by toxigenic *Corynebacterium ulcerans*, Norway. A) Pseudomembranes coughed up by the patient on day 1 of hospitalization. B) Pseudomembranes removed by bronchoscopy on day 4 of hospitalization. C) Trachea and carina after bronchoscopy on day 4 of hospitalization. D) Pseudomembranes removed from lower airways via bronchoscopy on day 4 of hospitalization.

An initial throat swab sample from hospitalization day 2 was *C. ulcerans*–negative, but follow-up samples from days 5 and 8 were *C. ulcerans*–positive. Follow-up samples on days 18 and 19 were negative, and the patient’s isolation was discontinued; he was discharged after 23 days. Comparison of antibody levels against diphtheria toxoid showed a protective level in 2020 (0.18 IU/mL) and >3.0 IU/mL on hospitalization day 7.

At 2-month follow-up, the patient had bilateral thigh numbness. Electroneurography confirmed sensorimotor polyneuropathy. 

Although severe *C. ulcerans* infections have been described (*3*,*4*), detailed clinical data on patients with positive blood cultures remain rare. Bacteremia appears linked to severe immunosuppression (*3*,*5*). This patient had protective diphtheria antibody titers but fulminant illness developed, requiring ventilation, antibiotics, and diphtheria antitoxin. A similar pattern was observed during a diphtheria epidemic in Russia in the 1990s (*6*). Although nontoxigenic *C. diphtheriae* can cause severe disease, diphtheria toxin remains the main virulence factor, inhibiting protein synthesis and causing cell death, especially in myocardium and peripheral nerves (*7*). This patient’s ventricular arrhythmia and subsequent polyneuropathy were consistent with diphtheria toxin–mediated toxicity (*7*).

Toxigenic *C. ulcerans* transmission remains uncertain. Although primarily zoonotic, with dairy cattle as a classic reservoir (*1*), infections from domestic and wild animals have been reported (*3*,*4*). We suspected but could not confirm transmission from this patient’s dog because no microbial samples were collected. The dog showed no signs of respiratory or skin disease before the patient’s hospitalization and remained in good health. 

Because *C. ulcerans* among animals is not notifiable in Norway, no official data or surveillance results are available. The sequence type 1032 variant had not been previously reported in humans and has since been identified in 1 other sample from another patient in Norway 6 months later; however, the 2 patients had no known epidemiologic link (V. Skogen, unpub. data). Whole-genome sequence analysis revealed substantial genomic differences between the 2 patients’ isolates, supporting separate infection sources. Because potential human-to-human transmission has been suggested (*8*–*10*), we applied isolation precautions, but no secondary cases were detected among close contacts or healthcare workers.

In summary, diagnosing diphtheria-like illness in low-prevalence settings is difficult because selective media are rarely used, and standard respiratory culture media might not detect *Corynebacterium* spp. bacteria. In this case, positive blood culture aided in early recognition, an uncommon but critical finding because classic diphtheria symptoms developed. This case underscores that *C. ulcerans* is an emerging zoonotic pathogen capable of causing life-threatening disease and highlights that early microbiological diagnosis is crucial, even in regions where diphtheria is rare.
